# The clinical and predictive value of ^18^F‐FDG PET/CT metabolic patterns in a clinical Chinese cohort with autoimmune encephalitis

**DOI:** 10.1111/cns.14821

**Published:** 2024-07-01

**Authors:** Yuwei Dai, Zehua Zhu, Yongxiang Tang, Ling Xiao, Xianghe Liu, Min Zhang, Bo Xiao, Kai Hu, Lili Long, Yuanyuan Xie, Shuo Hu

**Affiliations:** ^1^ Department of Neurology, Xiangya Hospital Central South University Changsha Hunan P.R. China; ^2^ National Clinical Research Center for Geriatric Diseases, Xiangya Hospital Central South University Changsha Hunan P.R. China; ^3^ Clinical Research Center for Epileptic disease of Hunan Province Central South University Changsha Hunan P.R. China; ^4^ Department of Nuclear Medicine, Xiangya Hospital Central South University Changsha Hunan P.R. China; ^5^ Key Laboratory of Biological Nanotechnology of National Health Commission, Xiangya Hospital Central South University Changsha Hunan P.R. China; ^6^ Division of Life Sciences and Medicine, Department of Nuclear Medicine, The First Affiliated Hospital of USTC University of Science and Technology of China Hefei Anhui P.R. China

**Keywords:** ^18^F‐FDG PET/CT, autoimmune encephalitis, diagnosis, prognosis

## Abstract

**Aims:**

To investigate the diagnostic and predictive role of ^18^F‐FDG PET/CT in patients with autoimmune encephalitis (AE) as a whole group.

**Methods:**

Thrty‐five patients (20 females and 15 males) with AE were recruited. A voxel‐to‐voxel semi‐quantitative analysis based on SPM12 was used to analyze ^18^F‐FDG PET/CT imaging data compared to healthy controls. Further comparison was made in different prognostic groups categorized by modified Rankin Scale (mRS).

**Results:**

In total, 24 patients (68.6%) were tested positive neuronal antibodies in serum and/or CSF. Psychiatric symptoms and seizure attacks were major clinical symptoms. In the acute stage, 13 patients (37.1%) demonstrated abnormal brain MRI results, while 33 (94.3%) presented abnormal metabolism patterns. ^18^F‐FDG PET/CT was more sensitive than MRI (*p* < 0.05). Patients with AE mainly presented mixed metabolism patterns compared to the matched controls, demonstrating hypermetabolism mainly in the cerebellum, BG, MTL, brainstem, insula, middle frontal gyrus, and relatively hypometabolism in the frontal cortex, occipital cortex, temporal gyrus, right parietal gyrus, left cingulate gyrus (*p* < 0.05, FWE corrected). After a median follow‐up of 26 months, the multivariable analysis identified a decreased level of consciousness as an independent risk factor associated with poor outcome of AE (HR = 3.591, *p* = 0.016). Meanwhile, decreased metabolism of right superior frontal gyrus along with increased metabolism of the middle and upper brainstem was more evident in patients with poor outcome (*p* < 0.001, uncorrected).

**Conclusion:**

^18^F‐FDG PET/CT was more sensitive than MRI to detect neuroimaging abnormalities of AE. A mixed metabolic pattern, characterized by large areas of cortical hypometabolism with focal hypermetabolism was a general metabolic pattern. Decreased metabolism of right superior frontal gyrus with increased metabolism of the middle and upper brainstem may predict poor long‐term prognosis of AE.

## Graphical abstract

The present study investigates diagnostic and predictive role of ^18^F‐FDG PET/CT in patients with autoimmune encephalitis (AE) as a whole group. Our findings suggest that ^18^F‐FDG PET/CT is more sensitive than MRI in the early diagnosis of AE. A mixed metabolic pattern, characterized by large areas of cortical hypometabolism with focal hypermetabolism is a general metabolic pattern. Decreased metabolism of right superior frontal gyrus with increased metabolism of the middle and upper brainstem may predict poor long‐term prognosis of AE.

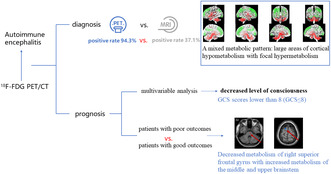

## 1 INTRODUCTION

Autoimmune encephalitis (AE) is a debilitating inflammatory brain disorder characterized by acute or subacute onset of neuropsychiatric symptoms.^1^ AE is associated with autoantibodies targeting against neuronal cell surface antigens, including synaptic receptors, neuronal cell‐surface proteins, or ion channels (eg, glioma inactivated 1 protein [LGI‐1], and N‐methyl‐D‐aspartate receptor [NMDAR]), or intracellular antigens (eg, glutamic acid decarboxylase 65‐kDa isoform [GAD65], Hu).^2^

Accurate diagnosis of AE is challenging because of the heterogeneity in clinical symptoms, low positive rate of laboratory testing tests, and lack of specificity in neuroimaging presentations.^3^ The clinical manifestations of AE are varied and non‐specific, including behavior problems, psychosis, seizures, and abnormal movements, often overlapped with infectious encephalitis, metabolic disease, and psychotic disorders.^4^ The early diagnosis of AE promotes earlier initiation of immunotherapy, which is a benefit for good prognosis.^5^, ^6^ Detecting neuronal antibodies in serum or cerebrospinal fluid (CSF) is critical to get a correct diagnosis. But antibody testing is not readily available in most hospitals, usually taking several weeks.^1^ Failure to detect known neural autoantibodies could not rule out the suspicion of AE, and those patients without neuronal antibodies were named antibody‐negative AE.^7^, ^8^

Clinical diagnostic criteria for AE developed by Graus et al. promoted the early diagnosis of AE and avoided delays in treatment caused by waiting for antibody test results.^1^ For those patients with high clinical suspicion of AE, these criteria required the combination of clinical history and auxiliary examination results, including electroencephalogram (EEG) and magnetic resonance imaging (MRI), regardless of the availability of antibody tests. In the workup of suspected AE, head MRI, as the most common imaging technique used in AE presented the limited value. The increased T2/fluid‐attenuated inversion recovery (T2/FLAIR) signal may involve multiple brain regions including cerebral cortex, cerebellum, striatum, brainstem with or without enhancement. Additionally, more than 50% of patients with AE demonstrated normal or nonspecific MRI results.^9^ There is an urgent need to identify a more sensitive neuroimage biomarker aiding the diagnosis of AE.

Recently, 2‐deoxy‐2‐^18^Ffluoro‐D‐glucose‐(^18^F‐FDG) positron emission tomography/computed tomography (PET/CT) imaging has been recognized as a potentially useful tool for diagnosing AE. Several studies have suggested ^18^F‐FDG PET/CT has higher sensitivity than MRI in assisting diagnosis.^10^

Solnes et al. found ^18^F‐FDG PET/CT was more often abnormal compared to patients with AE with MRI results during the diagnostic period (22/23, 95.6% vs. 10/23, 43%).^11^ Besides, some specific metabolic patterns associated with certain types of AE or special symptoms have been identified. For patients with anti‐NMDAR encephalitis, abnormalities in ^18^F‐FDG PET/CT were frontotemporal hypermetabolism and occipital hypometabolism, followed by metabolic normalization with recovery.^12^ Basal ganglia (BG) hypermetabolism implied the potential involvement with facio‐brachial dystonic seizures (FBDS).^13^ The decrease in the cortex/striatal metabolic ratio served as an imaging biomarker for the differentiation of patients with AE from mild cognitive impairment (MCI).^14^ However, the Graus criteria mentioned ^18^F‐FDG PET/CT as an alternative to MRI only for the definition of definite AE.^1^ The role of ^18^F‐FDG PET/CT in subjects with possible AE or probable AE needs more evidence.

Most previous works were restricted to delineate metabolic patterns on a specific autoantibody or a single syndrome, rather than on the whole AE group.^14^ In addition, brain PET images for semi‐quantitative analysis were extracted from whole‐body ^18^F‐FDG PET/CT, reducing the spatial resolution of imaging data.^12^ ^18^F‐FDG PET/CT studies on AE have been limited to visual interpretation or mingle with a certain proportion of patients after the acute stage of disease.^13^, ^15^ In the present study, we aimed to determine the brain metabolic patterns of glucose metabolism changes in a cohort of patients suffering from AE, including both antibody‐positive AE and antibody‐negative AE, in comparison to healthy controls matched on sex and age, using Statistical Parametric Mapping (SPM) analysis. Furthermore, we investigated the correlation of metabolism patterns with the condition of long‐term prognosis in patients with AE.

## 2 MATERIALS AND METHODS

### 2.1 Participants selection

A single‐center retrospective study was conducted at Xiangya Hospital, Central South University from January 2016 to December 2020. The study was approved by the Medical Ethics Committee of Xiangya Hospital, Central South University, and was carried out in compliance with the Declaration of Helsinki. Written Informed consent for inclusion in this study and publication for clinical details were obtained from each participant involved in this study.

**FIGURE 1:**
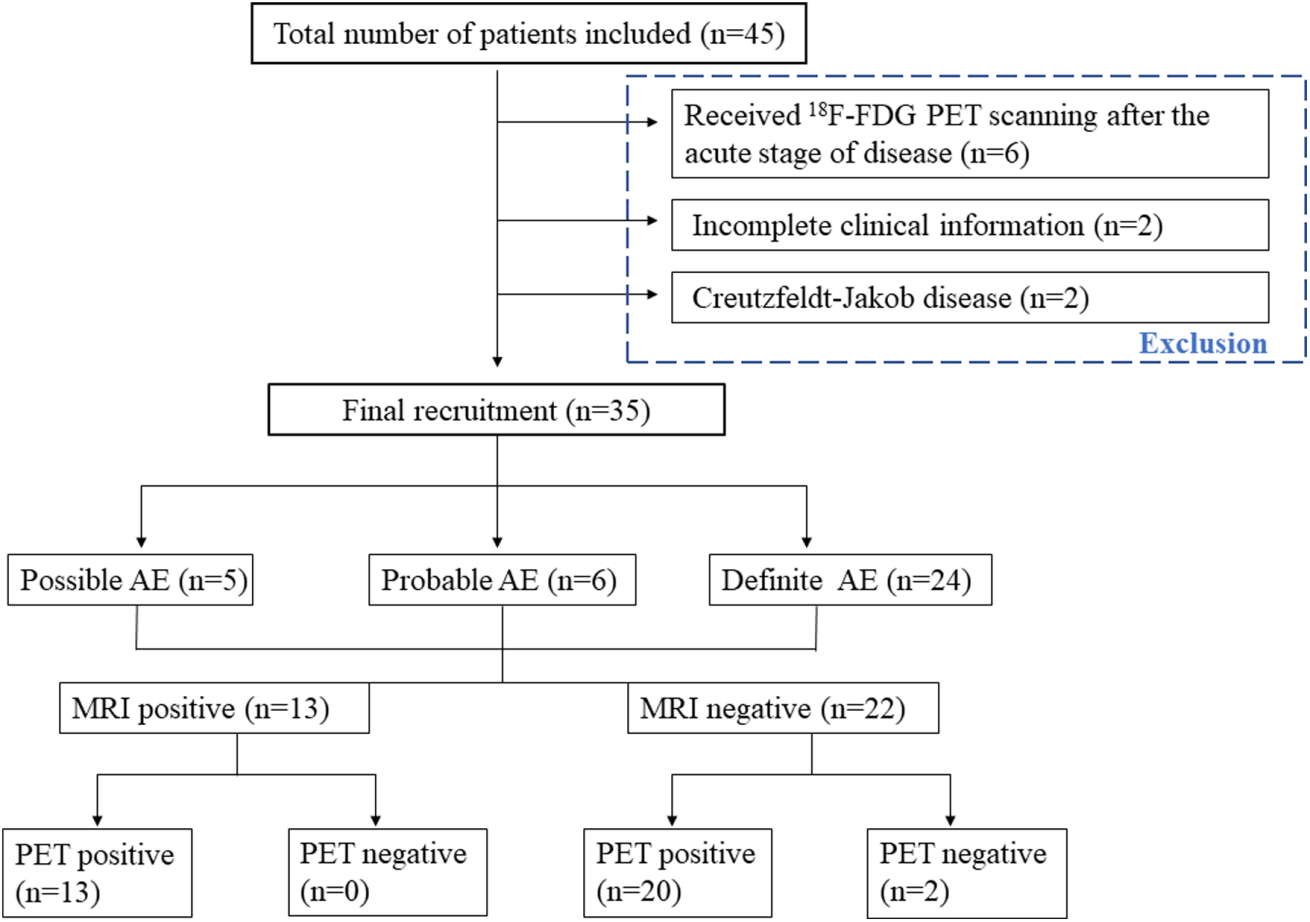
Flowchart of enrolled patients with autoimmune encephalitis.

During this period, a total of 340 adults diagnosed with suspected AE were referred to our hospital, of whom 45 patients were performed with cerebral ^18^F‐FDG PET imaging. After reviewing their electric medical recording carefully, 10 patients were excluded by (1) 6 patients who underwent PET scanning after the acute stage of disease; (2) 2 patients had incomplete clinical information; (3) 2 patients modified the final diagnosis with Creutzfeldt–Jakob disease. Finally, 35 patients with AE were recruited for this study (Figure 1).

The inclusion criteria were as follows: (1) subacute onset of neurological symptoms (working memory deficits, seizure, decreased level of conscience, movement disorders, unexplained dysautonomia, or abnormal movements) or psychiatric symptoms (behavior changes, disturbances in mood or character, hallucinations, speech dysfunction); (2) at least one of the following abnormalities: CSF with pleocytosis (white blood cell count of more than 5 cells per mm^3^), CSF oligoclonal bands or elevated IgG index, CSF elevated protein, abnormal EEG, MRI features suggestive of AE; (3) taking the PET examination in a relatively acute disease course, according to previous definition of the acute phase (within 3 months) and chronic phase (over 3 months) of AE^1^, ^13^; (4) reasonable exclusion of alternative causes.

All patients were further classified as (1) antibody‐positive AE based on Graus criteria^1^: autoimmune neuronal antibodies were detected in CSF and/or serum, including definite‐AE, antibody‐positive limbic encephalitis (Ab‐positive‐LE), and anti‐NMDAR encephalitis; (2) antibody‐negative AE: the diagnosis was made when all three of the following criteria have been met^16^, ^17^: (i) none of the known neuronal antibody was identified in the serum or CSF sampling; (ii) ≥1 feature present: CSF‐specific oligoclonal bands; intrathecal synthesis of immunoglobulin or elevated CSF IgG index; CSF pleocytosis (>5 cells/mm^3^) or elevated protein; brain MRI abnormalities suggestive of AE; effective immunotherapy; (iii) reasonable exclusion of alternative causes. These patients were sorted into Ab‐negative‐LE, probable‐AE, possible‐AE.^18^ Two experienced neurologists reviewed the medical record and made the corresponding classification (Yuwei Dai and Yuanyuan Xie).

The data concerning demographic information, clinical characteristics, associated neoplasia, treatment regimes, treatment delay as expressed in weeks between the onset of symptoms, and the duration of symptom onset and ^18^F‐FDG PET/CT were collected by reviewing patients' charts and databases. First‐line immunotherapy included intravenous methylprednisolone pulse (IVMP), intravenous immunoglobulin (IVIG), and plasma exchange (PLEX); rituximab (RTX) and cyclophosphamide served as second‐line immunotherapy. The levels of consciousness of enrolled patients were evaluated based on the Glasgow Coma Scale (GCS) score on admission. GCS scores lower than 8 (GCS≤8) indicated decreased level of consciousness.^19^ Forty‐three age‐matched, gender‐matched volunteers without any neurological or psychiatric illness were enrolled as healthy controls.

### 2.2 Exclusion of infectious etiologies

Infectious evaluations of serum and CSF were performed for all patients as a diagnostic procedure of AE. The following measures were adopted to exclude possible infectious etiologies: Gram stain and India ink stain; aerobic and anaerobic bacterial cultures; mycobacterium culture and acid‐fast bacillus stain; fungal culture; immunoglobulin M (IgM) and IgG specific for parasites; polymerase chain reaction (PCR) tests for enterovirus, Epstein Barr virus (EBV), herpes simplex virus (HSV) 1 and 2, varicella‐zoster virus, or human herpes virus 6. No definite evidence of infection was found in these enrolled cases.

### 2.3 Neuronal antibody detection

For every patient with suspected AE, comprehensive neural antibody studies were conducted in serum and CSF, including both indirect immunofluorescence assay (IIFA) fixed by rat brain tissues for unknown antibodies and commercial workup panels for known antibodies.

First, serum and CSF samples were screened with indirect tissue‐based assays (TBAs) composed of rat cerebellum and rat hippocampus for the presence of neuronal antibodies. Then, those patients were evaluated for known antibodies in the serum and CSF, using IIFA fixed by commercial transfected HEK293 cells assays (CBAs) to test antibodies against cell surface antigens (Euroimmun, Lübeck, Germany), including NMDAR, LGI‐1, contactin‐associated protein‐2 (CASPR2), α‐amino‐3‐hydroxy‐5‐methyl‐4‐isoxazole propionic acid receptor (AMPAR), γ‐aminobutyric acid type B receptor (GABA_B_R) and IgLON family member 5 (IgLON5), and immunoblotting (Euroimmune Ag) to detect intracellular antibodies (GAD65, SOX1, Hu, Yo, Ri, Ma2, CV2, amphiphysin). Positivity was determined by repeat testing (≥2 times per sample). The antibody testing results were interpreted by two experienced investigators using the signal of the surrounding fields as negative controls.^20^

### 2.4 Evaluation and follow‐up of long‐term prognosis

Two neurologists (Xianghe Liu and Lili Long) retrospectively evaluated the severity of clinical symptoms by a comprehensive scale proposed recently, the Clinical Assessment Scale in Autoimmune Encephalitis (CASE).^21^ Meanwhile, the modified Rankin Scale (mRS) was also conducted to evaluate the neurological disability of all patients.^22^ The CASE score (range: 0–27) and mRS score (range: 0–6) were obtained at four time points (at admission, at disease peak, at discharge, and at the last follow‐up) based on medical records. Responders to treatment were defined as the decrease of at least 1 point in mRS score after immunotherapy.^18^

The follow‐up was achieved via subsequent outpatient visits. The long‐term outcomes were evaluated at least 1 year from disease onset.^6^ Patients were categorized into two groups based on the mRS score at the last follow‐up: good outcome (mRS: 0–2) and poor outcome (mRS: 3–6).

### 2.5 Review of brain MRI

Patients' brain MRIs were collected using 3.0‐Tesla or 1.5‐Teala MAGNETOM Verio scanner (Siemens, Germany) in Xiangya Hospital, Central South University. The following sequences were acquired: T1‐weighted images (T1WI), T2‐weighted images (T2WI), fluid‐attenuated inversion recovery (FLAIR), diffusion‐weighted images (DWI), and gadolinium‐enhanced images. In the case of multiple MRI examinations, MRI data interpretation primarily focused on the results nearest to the onset of symptoms. MRI images with evidence of underlying encephalitis or inflammatory changes (T2 or T2/FLAIR hyperintensities restricted to one or both mesial temporal lobes, or multifocal in gray matter, white matter, or both in the cerebral cortex, cerebellum, striatum, brainstem) were considered as positive. Blinded interpretation of MRI results was conducted by two experienced radiologists.

### 2.6 ^18^F‐FDG PET/CT scanning

All subjects accepted ^18^F‐FDG PET/CT scanning at the PET Center of Xiangya Hospital, Central South University using a Discovery Elite PET/CT scanner (GE Healthcare, Boston, MA, United States) during the acute phase of the disease. All participants were required to fast for at least 6 h and discontinue all steroid hormones or antiseizure drugs (ASDs) for at least 24 h. Fasting blood glucose levels of all patients did not exceed 8 mmol/L before injection of ^18^F‐FDG. ^18^F‐FDG was intravenously injected following a dose of 3.7 MBq/kg within 1 min. Before PET scan acquisition, participants were allowed to rest quietly in a dedicated room for an hour. A low‐dose CT scan (120 kV, 60–180 mA/s, slice thickness 3.75 mm) was started first, brain PET scanning was performed for 15–20 min followed by whole‐body PET scanning for 35–45 min. The whole‐body PET scans ranged from the top of the head down to mid‐thigh, to screen occult tumors. The full width of the scan at half maximum was 5.4 mm. The brain images were reconstructed into a 192 × 192 trans‐axial matrix (35 cm field of view) using the 3D VUE Point (GE Healthcare) ordered‐subset expectation–maximization algorithm. CT images were obtained for attenuation correction simultaneously.

### 2.7 ^18^F‐FDG PET/CT imaging analysis

PET imaging data were analyzed by SPM12 software (Welcome Department of Cognitive Neurology, London, United Kingdom), implanted in MATLAB 2018a (MathWorks Inc., Sherborn, MA, USA) environment. The image preprocessing steps were as follows: scans from all individuals were firstly segmented and spatially normalized into standard stereotactic Montreal Neurological Institute (MNI) space with voxel sizes of 2 mm × 2 mm × 2 mm. Then, normalized images were smoothed by an 8‐mm Full‐Width Half‐Maximal (FWHM) 3D Gaussian kernel to increase the signal‐to‐noise ratio. Subsequently, PET image values were processed to intensity normalization, performed by proportional scaling to overall activity.

Once the PET images were preprocessed, a two‐sample *t*‐test was conducted to delineate metabolic pattern characteristics of single subjects with age and gender as the nuisance variables, comparing each patient with the control group. FDG uptake was compared voxel‐to‐voxel between patients and the control group using two‐*t*‐sample‐test of SPM. We also compared AE patients with good outcomes between AE patients with poor outcome using the same way to the predictive value of ^18^F‐FDG‐PET biomarkers on long‐term prognosis. Metabolic changes were considered statistically significant at the strict threshold (*p* < 0.001) and corrected for multiple comparisons (family‐wise error [FWE] corrected, *p* < 0.05) with cluster size (K_E_) above 20 contiguous voxels. If significant metabolic changes were not found, a more lenient threshold was set (*p* < 0.001, uncorrected; K_E_ = 20 voxels).

### 2.8 Statistical analysis

Continuous variables were described using means ± standard deviation (SD) or median (interquartile range [IQR]). The counting data were presented as percentages (%). Continuous variables were tested to determine whether they conformed to a normal distribution by the Shapiro–Wilk normality test first. For the univariate analysis of poor long‐term outcomes, continuous variables were compared using the *t*‐test or non‐parametric Mann–Whitney *U*‐test, and Fisher's exact tests were used for categorical variables. The variables with *p* values < 0.05 on univariate analysis were further selected by the least absolute shrinkage and selection operator (LASSO) regression using the R software package glmnet.^23^ The features selected by LASSO regression were subsequently entered into the multivariate Cox proportional hazards regression model. The strength of association was evaluated by Hazard ratios (HR) and corresponding 95% confidence interval (CI) in the multivariate Cox regression model. A two‐tailed *p*‐value < 0.05 was considered significant. Statistical analyses were performed with SPSS 26.0 software package for Windows (IBM Corp.) and R language (version 4.3.3).

## 3 RESULTS

### 3.1 Clinical characteristics

Demographic information and clinical characteristics of 35 patients (15 males, 20 females) with AE are summarized in Table 1. The average age of disease onset was 43.89 ± 18.21 years old. Forty‐three age‐matched healthy subjects (mean age: 45.13 ± 16.25 years old, 20 females) served as controls.

**TABLE 1:** Demographic and clinical characteristics of patients with autoimmune encephalitis.

Variables	Values
Total (*n*)	35
Age at onset, years	43.89 ± 18.21
Sex, female, *n* (%)	20/35 (57.1%)
Clinical symptoms, *n* (%)	
Seizures	20/35 (57.1%)
Psychiatric symptoms	19/35 (54.3%)
Decreased level of consciousness	8/35 (22.9%)
Memory deficit	6/35 (17.1%)
Central hypoventilation	5/35 (14.3%)
Ataxia	3/35 (8.6%)
Dystonia	3/35 (8.6%)
Speech dysfunction	2/35 (5.7%)
Tumors, *n* (%)	5/35 (14.3%)
Autoantibodies, *n* (%)	
Positive	24/35 (68.6%)
Against cell surface antigens, *n* (%)	21/35 (60.0%)
NMDAR	10/35 (28.6%)
GABA_B_R	5/35 (14.3%)
CASPR2	2/35 (5.7%)
LGI‐1	2/35 (5.7%)
D2R	1/35 (2.9%)
IgLON5	1/35 (2.9%)
Against intracellular antigens, *n* (%)	3/35 (8.6%)
SOX1	2/35 (5.7%)
GAD65	1/35 (2.9%)
Negative	11/35 (31.4%)
CSF findings	35/35 (100%)
Normal finding	21/35 (60.0%)
Increased intracranial pressure^a^	8/35 (22.9%)
Pleocytosis^b^	12/35 (34.3%)
Elevated protein levels	9/35 (25.7%)
Oligoclonal bands	15/25 (60%)
EEG monitoring, *n* (%)	20/35 (57.1%)
Abnormal	17/20 (85.0%)
Brain MRI findings	35/35 (100%)
Normal or non‐specific results	22/35 (62.9%)
Abnormal findings	13/35 (37.1%)
ICU administration	8/35 (22.9%)
Immunotherapy, *n* (%)	35/35 (100%)
Combined with second‐line immunotherapy	11/35 (31.4%)
Response to treatment	21/35 (60.0%)
Relapse, *n* (%)	6/35 (17.1%)
Interval between symptoms onset and the first relapse, median (range), months	15.5 (IQR: 9.5–33)
mRS at admission, median (IQR)	5.0 (4–5)
mRS at disease peak, median (IQR)	5.0 (4–5)
mRS at discharge, median (IQR)	3.0 (1–4)
mRS at the last follow‐up, median (IQR)	2.0 (1–4)
CASE score	
CASE at admission, median (IQR)	5.0 (4–7)
CASE at disease peak, median (IQR)	7.0 (6–9)
CASE at discharge, median (IQR)	4.0 (3–6)
CASE at the last follow‐up, median (IQR)	3.0 (1–6)^c^
Follow‐up and outcomes	35/35 (100%)
Interval between last follow‐up and disease onset, median (IQR), months	26 (20–40)
Favorable long‐term outcome	18/35 (51.4%)

Abbreviations: CASE, Clinical Assessment Scale in Autoimmune Encephalitis; CASPR2, contactin‐associated protein‐2; CSF, cerebrospinal fluid; D2R, dopamine D2 receptor; EEG, electroencephalogram; GABA_B_R, Gamma‐aminobutyric acid type B receptor; GAD65, glutamic acid decarboxylase 65; ICU, intensive care unit; IgLON5, IgLON family member 5; IQR, interquartile range; LGI‐1, leucine‐rich glioma inactivated 1; MRI, magnetic resonance imaging; mRS, modifies Rankin Scale; NMDAR, N‐methyl‐D‐aspartate receptor.

^a^ Intracranial pressure >180 mmH_2_O.

^b^ White blood cell counts of more than five cells per mm^3^.

^c^ Not available CASE score for four dead patients at the last follow‐up.

In the acute stage of AE, the main symptoms of this cohort were seizures (20/35, 57.1%), psychiatric symptoms (19/35, 54.3%), followed by decreased level of consciousness (8/35, 22.9%), memory deficit (6/35, 17.1%), central hypoventilation (5/35, 14.3%). Other clinical symptoms included ataxia (3/35, 8.6%), dystonia (3/35, 8.6%), and speech dysfunction (2/35,5.7%). 2 subjects presented with FBDS, and they were tested positive for LGI1 antibodies. Tumors were found in 5 patients (2 with lung cancer, 1 with colon cancer, 1 with hematological malignancies, and 1 with breast cancer).

All patients had tested antibodies in both serum and CSF (35/35, 100%). Positive neural antibodies were detected in 24 patients (24/35, 68.6%), and 11 patients were classified as antibody‐negative AE (11/35, 31.4%). Twenty‐four patients with positive neural antibodies were further classified into 10 anti‐NMDAR encephalitis, 3 Ab‐positive‐LE, and 11 definite AE. In 24 patients with antibody‐positive AE, 21 patients (21/35, 60.0%) found positive neuronal antibodies in both serum and CSF (10 NMDAR; 5 GABA_B_R; 2 LGI1;2 CASPR2; 1 D2R; 1 IgLON5), 3 patients (3/35, 8.6%) only tested positive intracellular antibodies in serum (2 SOX; 1GAD65). In 11 patients with antibody‐negative AE, 6 were probable AE and 5 were possible AE. A detailed table about the clinical, laboratory, and radiological features of patients with antibody‐negative AE is in Table S1.

CSF samples were collected during hospitalization (35/35, 100%). Eight patients had increased intracranial pressure (≥180 mmH_2_O). White blood cell (WBC) counts in the CSF of 12 subjects were mildly increased (12/35, 34.3%). 9 (9/35, 25.7%) had elevated cerebrospinal fluid protein levels CSF protein. Among 25 patients who were tested for CSF oligoclonal bands, 60% (15/25, 60.0%) reported a positive result. Twenty patients completed 24‐h dynamic or video EEG monitoring (20/35, 57.1%), and 17 showed abnormal EEG results, mainly revealing slow‐waves or slow‐sharp waves (17/20, 85.0%).

All subjects were treated with first‐line immunotherapy (35/35,100.0%). Eleven of them received additional second‐line immunotherapy based on first‐line immunotherapy during the acute phase (11/35, 31.4%). Twenty‐one patients (21/35, 60.0%) had a good response to treatment. Six patients experienced at least one relapse (6/35, 17.1%), of which 4 were antibody‐positive AE. The median interval between disease onset and first recurrence was 15.5 months (IQR: 9.5–33). The median mRS scores at admission, at disease peak, at discharge, at the last follow‐up were 5.0, 5.0, 3.0, and 2.0. The median CASE scores at admission, at disease peak, at discharge, at the last follow‐up were 5.0, 7.0, 4.0, and 3.0.

### 3.2 PET imaging analysis

The details of imaging results (both MRI and ^18^F‐FDG PET/CT) for individual patients were summarized in Table S2. The MRIs were performed at a median of 17 days (IQR: 12.0–33.0) after disease onset and 23 days (IQR:15.0–35) for ^18^F‐FDG PET/CT. The median time interval between MRI and PET was 3 days (IQR:3.0–5.0). There was no difference in time interval from the initial symptom onset until imaging examination. For MRI imaging results, 22 individuals showed normal or unspecific MRI results (22/36, 62.9%). Abnormal findings were observed in 13 patients (13/35, 37.1%), in which 3 patients presented with abnormal T2/FLAIR signal change in the bilateral medial temporal lobe (MTL). Meanwhile, 33 patients presented abnormal metabolism patterns on ^18^F‐FDG PET/CT (33/35, 94.3%). ^18^F‐FDG PET/CT demonstrated higher sensitivity than MRI (94.3% vs. 37.1%, *p* < 0.05). In particular, the detection rate of ^18^F‐FDG PET/CT was 90.9% in patients with normal MRI (20/22, 90.9%), and 13 patients with abnormal MRI results also detected abnormal brain metabolism.

**FIGURE 2:**
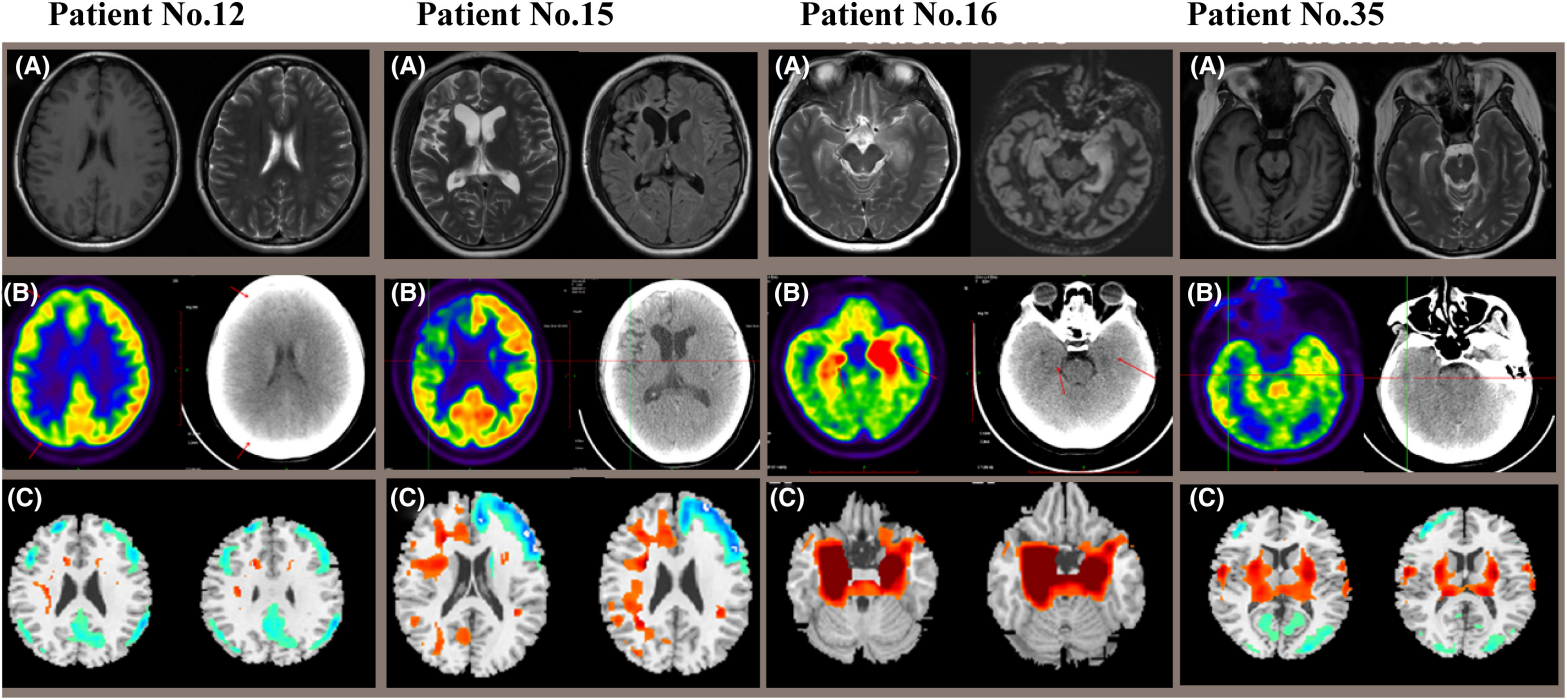
Corresponding MRI images, abnormal cerebral glucose metabolism patterns and ^18^F‐FDG PET/CT results based on SPM of 4 patients with AE. (A) MRI imaging, (B) cerebral glucose metabolism patterns, (C) ^18^F‐FDG PET/CT results based on SPM (*p* < 0.05, FWE corrected). The hypermetabolism regions were shown in orange‐red color, and hypometabolism regions were shown in blue‐green color. ^18^F‐FDG PET/CT, 2‐deoxy‐2‐^18^Ffluoro‐D‐glucose‐(^18^F‐FDG) positron emission tomography/computed tomography; AE, autoimmune encephalitis; FWE, family‐wise error; MRI, magnetic resonance imaging; SPM, Statistical Parametric Mapping.

**FIGURE 3:**
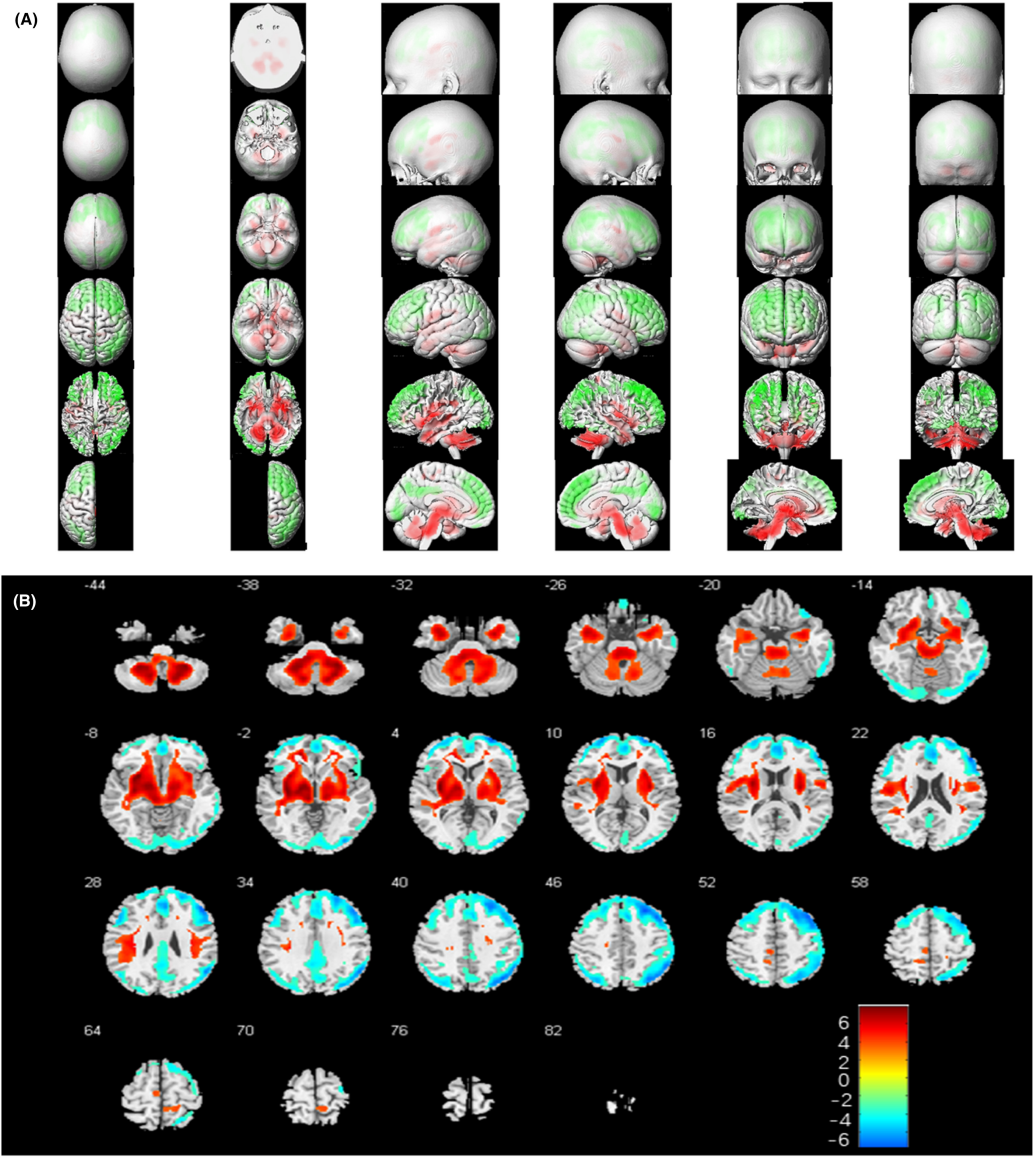
Groupwise analysis of ^18^F‐FDG PET/CT metabolic patterns between patients with AE and healthy controls based on SPM (*p* < 0.05, FWE corrected). Slice view (A) and render view (B) were created by xjView toolbox (https://www.alivelearn.net/xjview/). The hypermetabolism regions were shown in orange‐red color, and hypometabolism regions were shown in blue‐green color. ^18^F‐FDG PET/CT, 2‐deoxy‐2‐^18^Ffluoro‐D‐glucose‐(^18^F‐FDG) positron emission tomography/computed tomography; AE, autoimmune encephalitis; FWE, family‐wise error; SPM, Statistical Parametric Mapping.

Figure 2 demonstrated corresponding MRI images, abnormal cerebral glucose metabolism patterns, and ^18^F‐FDG PET/CT results based on SPM12 of 4 representative patients (No. 12, No. 15, No. 16, No. 35). Compared to the healthy control group, brain metabolic images of each subject were performed with semi‐quantitatively analysis (*p* < 0.05, FWE corrected). Thirty‐three patients presented mixed metabolism patterns, showing cortical hypometabolism combined with focal hypermetabolism (33/35, 94.3%). For groupwise analysis, patients with AE mainly presented mixed metabolism patterns compared to the matched controls, demonstrating hypermetabolism of cerebellum, BG, MTL, midbrain, pons, medulla oblongata, insula, middle frontal gyrus, paracentral lobule, precuneus, precentral gyrus and relatively hypometabolism in the frontal cortex, occipital cortex, middle temporal gyrus, superior temporal gyrus, right superior parietal gyrus, middle parietal gyrus, precuneus, left cingulate gyrus (*p* < 0.05, FWE corrected; Figure 3). Mixed metabolism patterns were a representative feature of patients with AE in the acute stage.

### 3.3 Group analyses of individual patients in different long‐term outcome subgroups

#### 3.3.1 Univariate analysis of predictors of poor long‐term outcome

In this study, after the median follow‐up time of 26.0 months (IQR: 20.0–40.0) since disease onset, the median score on the mRS scale was 2 points (IQR:1–4). A good outcome was achieved in 18 patients (18/35, 51.4%), while 17 showed a poor long‐term outcome (17/35, 48.6%), in which 4 patients died (4/35, 11.4%). The median interval between the last follow‐up and disease onset of patients with good outcome was 30.5 months (IQR:13.5–46.5), while 25.0 months (IQR: 20.5–39.5) in the group with bad outcome. We found no significant statistical difference between the two subgroups (*p* = 0.590) during the follow‐up interval of this study.

The comparison of demographic and clinical information among two subgroups is shown in Table 2. The mean age of patients with poor long‐term outcome was 50.76 ± 18.4 years, whilst the mean age of the good long‐term outcome group was 37.39 ± 15.9 years (*p* = 0.028). The rate of decreased level of consciousness in the good and poor outcome groups was 1/18 (5.6%) and 7/17 (41.2%), respectively (*p* = 0.018). Besides, 29.4% (5/17) experienced central hypoventilation in poor outcome group, which was significantly higher than that in the good outcome group (0/18, *p* = 0.019). Patients with poor outcomes also had a higher tumor incidence than the good outcome group (*p* = 0.019). The poor outcome group tended to have a lower percentage of receiving second‐line immunotherapy, compared with good outcome group (*p* = 0.027). There was no statistical significance in gender composition, some primary clinical symptoms (seizure, psychiatric symptoms, ataxia), and the rate of intensive care unit (ICU) admission (all *p* > 0.05). On antibody examination, antibody positive detection rate, or antibodies against intracellular antigens positive detection rate did not show any statistical differences among the two subgroups (both *p* > 0.05). Both groups did not differ in their mRS scores and CASE scores at disease peak (both *p* > 0.05). No difference was found in the interval from symptoms onset to initiation of immunotherapy (*p* = 0.351) or the proportion of first‐line immunotherapy (*p* = 1.000). There was no statistically significant difference in the time interval between the two groups receiving PET examinations (*p* = 0.424). The proportion of steroids or sedatives usage before PET scanning in the two subgroups also had no statistical difference (both *p* > 0.05). The rate of relapse or median time to relapse was similar in patients with good outcome and patients with poor outcome (both *p* > 0.05). Older age, reduced consciousness, central hypoventilation, tumors, and less usage of second‐line immunotherapy were more likely to be at risk of poor prognosis.

**TABLE 2:** Comparison of basic information between patients with AE with good long‐term outcome and poor long‐term outcome.

Variables	Good long‐term outcome (mRS: 0–2), *n* = 18	Poor long‐term outcome (mRS: 3–6), *n* = 17	*p* value
Interval between last follow‐up and disease onset, median (IQR), months	30.5 (13.5–46.5)	25.0 (20.5–39.5)	0.590^a^
Age at onset, average ± SD, years	37.39 ± 15.9	50.76 ± 18.4	0.028^a^ ^,^*
Female, (*n*%)	12/18 (66.7%)	8/17 (47.1%)	0.315^b^
Main clinical symptoms, (*n*%)			
Seizures	11/18 (61.1%)	9/17 (52.9%)	0.738^b^
Psychiatric symptoms	9/18 (50%)	10/17 (58.8%)	0.738^b^
Decreased level of consciousness	1/18 (5.6%)	7/17 (41.2%)	0.018^b^ ^,^ *
Central hypoventilation	0/18 (0%)	5/17 (29.4%)	0.019^b^ ^,^ *
Ataxia	1/18 (5.6%)	2/17 (11.8%)	0.603^b^
Tumors, (*n*%)	0/18 (0%)	5/17 (29.4%)	0.019^b^ ^,^ *
Admission to ICU, (*n*%)	3/18 (16.7%)	5/17 (29.4%)	0.443^b^
Abnormal brain MRI, (*n*%)	8/18 (44.4%)	5/17 (29.4%)	0.489^b^
Antibodies detection, positive, (*n* %)	12/18 (66.7%)	12/17 (70.6%)	1.000^b^
Antibodies against intracellular antigens, (*n*%)	1/12 (8.3%)	2/12 (16.7%)	1.000^b^
mRS at disease peak, median (IQR)	5 (3–5)	5 (5–5)	0.405^a^
CASE score at disease peak, median (IQR)	6.5 (5–10)	7 (6–9)	0.636^a^
Interval between disease onset and immunotherapy initiation, Median (IQR), days	21.5 (16.5–45.0)	32.0 (20.5–45.5)	0.351^a^
First‐line immunotherapy initiation, %	18/18 (100%)	17/17 (100%)	1.000^b^
Combined with second‐line immunotherapy, %	9/18 (50%)	2/17 (11.8%)	0.027^b^ ^,^ *
Interval between symptoms onset and ^18^F‐FDG‐PET, median (IQR), days	18.5 (13.8–35.8)	24.0 (17.5–39.5)	0.424^a^
Treated with corticosteroids before ^18^F‐FDG‐PET, %	5/18 (27.8%)	2/17 (11.8%)	0.402^b^
Treated with sedatives before ^18^F‐FDG‐PET, %	5/18 (27.8%)	5/17 (29.4%)	1.000^b^
Relapse, %	2/18 (11.1%)	4/17 (23.5%)	0.402^b^
Interval between symptoms onset and the first relapse, median (range), months	34 (32–36)	10.5 (8–20)	0.064^a^

Abbreviations: AE, autoimmune encephalitis; mRS, modifies Rankin Scale; CASE, Clinical Assessment Scale in Autoimmune Encephalitis; IQR, interquartile range; SD, standard deviation; ICU, intensive care unit; MRI, magnetic resonance imaging; ^18^F‐FDG PET/CT, 2‐deoxy‐2‐18Ffluoro‐D‐glucose‐(^18^F‐FDG) positron emission tomography/computed tomography.

* *p* < 0.05.

^a^ *t*‐Test or Mann–Whitney *U*‐test was used for continuous variables.

^b^ The Fisher's exact tests were used for categorical variables.

#### 3.3.2 Multivariate analysis of predictors of poor long‐term outcome

Five features identified by univariate analysis were further selected by LASOO regression. Three variables (decreased level of consciousness, tumors, combined with second‐line immunotherapy) selected by LASSO regression were finally included in a multivariate Cox regression model. These results were presented in Table 3. The multivariate analysis results showed that decreased level of consciousness was independently associated with poor outcome of AE (HR = 3.591, 95% CI = 1.272–10.375; *p* = 0.016). No significant association was found in other predictors of poor prognosis, including tumors or combined with second‐line immunotherapy (*p* > 0.05).

**TABLE 3:** Multivariate analysis of risk factors associated with poor long‐term outcome in patients with autoimmune encephalitis.

Variables	Hazard Ratios	95% CI	*p* value
Decreased level of consciousness	3.591	1.272–10.375	0.016*
Tumors	1.957	0.646‐5.929	0.235
Combined with second‐line immunotherapy	2.717	0.582–12.680	0.204

* *p* < 0.05.

#### 3.3.3 Prognostic value of PET imaging

**FIGURE 4:**
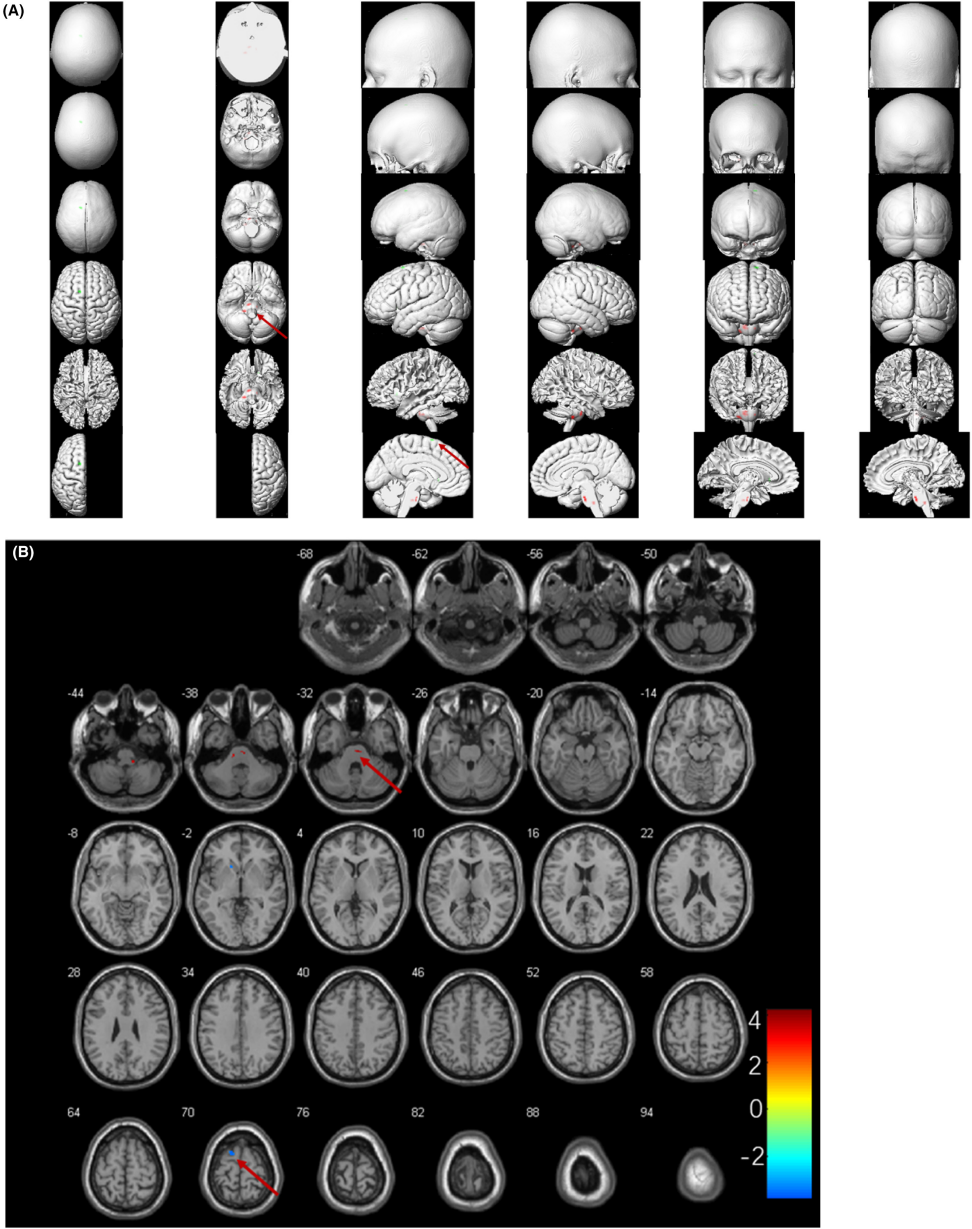
Metabolic results of two‐sample *t* test group analysis between two different prognostic subgroups (*p* < 0.001, uncorrected). Slice view (A) and render view (B) were created by xjView toolbox (https://www.alivelearn.net/xjview/). The hypermetabolism regions were shown in orange‐red color, and hypometabolism regions were shown in blue‐green color.

Further semi‐quantitative group analysis of metabolic patterns based on SPM found that patients with AE with poor outcomes showed decreased metabolism of the right superior frontal gyrus along with increased metabolism of the middle and upper brainstem, mainly located in bilateral pons, compared with those with good outcomes (*p* < 0.001, uncorrected, Figure 4).

## 4 DISCUSSION

In this present study, we retrospectively investigated cerebral metabolic patterns of 35 patients with AE in the acute stage based on a voxel‐based analysis of SPM, then we searched candidate neuroimage prognostic biomarkers of AE. Rather than limiting our focus to a specific antibody‐mediated encephalitis, we focused the identification of AE as a whole, not only those with antibody‐positive AE but also those with antibody‐negative AE. There were three significant implications of our study, first, in comparison with MRI, ^18^F‐FDG PET/CT might be more often abnormal sensitive in the acute stage of AE; then, a mixed metabolic pattern, characterized by large areas of cortical hypometabolism with focal hypermetabolism, was a typical finding of AE in acute stage. Lastly, decreased metabolism of right superior frontal gyrus along with increased metabolism of the middle and upper brainstem might be associated with a poor prognosis for patients with AE. These results provided clues for the application of ^18^F‐FDG PET/CT in the diagnosis and clinical management of AE.

For AE, as a group of complicated and treatable neuroinflammation conditions mediated by autoimmune antibodies, early diagnosis is essential to prompt initiation of immunotherapy thus generating a positive outcome.^24^ Although a clinical approach to diagnosis AE has emerged recently,^1^ many patients with suspected AE might be misdiagnosed first as “psychiatric patients” and miss the optimal timing for intervention, mostly due to the presence of antibody‐negative patients with AE or delay of antibody testing.^25^, ^26^ Besides, most patients with AE were clinically characterized by various and unspecific symptoms with normal or non‐specific findings of serum or CSF sampling, EEG and MRI, both in previous reports and our reports.^14^ As a major diagnostic imaging tool, the diagnostic value of MRI was limited. In our report, the proportion of MRI negative was as high as 62.9% as in previous reports.^4^ Probasco et al. also found that only 40% of patients with AE had consistent abnormal MRI findings.^27^

Numerous studies began to focus on the diagnostic value of ^18^F‐FDG PET/CT on AE.^11^ It has been widely reported that PET was more sensitive to detecting abnormal findings in AE than MRI.^28^ A recent systematic review encompassed 21 publications and 444 patients with AE, from which 87% (80–92%) showed alterations in brain FDG–PET images. The high detection sensitivity of ^18^F‐FDG PET/CT was in concordance with our results (94.3%).^10^ For 13 patients with abnormal MRI, lesions confirmed by MRI roughly corresponded to lesions with glucose metabolic abnormalities from PET images, indicating a better consistency between MRI and ^18^F‐FDG PET/CT. 94.3% of patients presented a mixed metabolic pattern, characterized by large areas of cortical hypometabolism with focal hypermetabolism. The discrepancy between MRI and ^18^F‐FDG PET/CT in detection rates of AE might be explained by neuronal dysfunction in the absence of structural alteration.^29^

The usefulness of brain ^18^F‐FDG PET/CT imaging has been acknowledged by the latest diagnostic criteria of LE. The guideline stated bilateral FLAIR/T2 abnormalities of MTL were required, and ^18^F‐FDG PET/CT hypermetabolism in bilateral MTL could serve as an alternative in the absence of such findings.^27^ A prospective study enrolled 104 pediatric patients with suspected AE and concluded brain ^18^F‐FDG PET/CT had higher specificity, sensitivity, and accuracy for diagnosis of AE in clinically suspected AE children. Nearly 60% of children with AE showed extensive hypometabolism in more than one lobe or one lobe with focal hypermetabolism, which supported evidence for the diagnosis in children with suspected AE.^16^ Mixed metabolic patterns may become common features of AE in both adults and children. Moreover, a systematic review tried to clarify distinct metabolic patterns of ^18^F‐FDG PET/CT among different AE subtypes, mainly focusing on anti‐NMDAR encephalitis and anti‐LGI1 encephalitis.^30^ In anti‐NMDAR encephalitis, PET findings revealed hypermetabolism in temporal lobe with/without metabolic alteration in cortical regions.^3^, ^30^ Mixed patterns of hyper‐ and hypo‐metabolism, characterized by frontal lobe hypometabolism combined with temporal lobe and right cerebellum hypermetabolism were detected in patients with anti‐NMDAR encephalitis of our cohort. The brain metabolism patterns of anti‐NMDAR encephalitis based on SPM in our cohort were shown in Figure S1. However, regarding the expensive cost and radioactivity of ^18^F‐FDG PET/CT, we proposed that brain ^18^F‐FDG PET/CT could be considered as a diagnostic tool under the following conditions: highly suspected AE but no positive paraclinical findings or paraneoplastic syndromes suggesting for the presence of tumors.

The proportion of good prognosis in our present study was 51.4%. Similarly, a recent multi‐center study conducted in Eastern China revealed that good clinical outcomes were achieved in 117 (63.24%) AE patients with a follow‐up period of at least 1 year.^31^ In our report, we have identified five variables associated with the long‐term outcome of AE using a univariate analysis: age, presence of potential neoplasm, level of consciousness, central hypoventilation, and the use of second‐line immunotherapy. However, further multivariate analysis showed that only a decreased level of consciousness was identified as an independent predictive risk factor. Chi et al. also found that a GCS score ≤8 at admission was significantly associated with an increased risk of mortality in patients with anti‐NMDAR encephalitis.^19^ Those with reduced level of consciousness were more likely to have a combination of severe complications, such as pneumonia and some infections, which might prolong recovery time.^32^ There is still no unified conclusion on the clinical variables affecting AE prognosis, heterogeneous study design, or different criteria for judging ending points produced conflicting results.^13^ In our study, patients with good outcome tended to have shorter interval between disease onset and immunotherapy initiation and to receive more second‐line immunotherapy, even if these variables did not reach statistical significance. This indicated that neurologists should initiate immunotherapy in patients with suspected AE as soon as possible.

Previous studies have tried to find representative neuroimage biomarkers serving as prognostic factors of AE, mostly focusing on MRI findings. In a case series of 41 patients with AE, Byun et al. found that normal MRI presentation was associated with seizure remission in half a year.^33^ It was also reported that diffuse cerebellar atrophy revealed by MRI often indicated a poor prognosis of anti‐NMDAR encephalitis.^34^ To our knowledge, less research has focused on the application of ^18^F‐FDG PET as a predictive biomarker to predict the long‐term outcome of AE. Among patients with anti‐NMDAR encephalitis, marked medial and lateral occipital lobe hypometabolism was more evident in those with severe neurologic disability compared with those less neurologically disabled.^35^ Recently, Liu et. al proposed that MTL hypermetabolism might serve as a prognostic biomarker in anti‐GABA_B_R encephalitis.^21^ In our cohort, decreased metabolism of the right superior frontal gyrus along with increased metabolism of the middle and upper brainstem has a relationship with poor clinical long‐term outcome in patients with AE. Prior studies revealed an important role that the upper brainstem played in consciousness maintenance, arousal, and attention.^36^ Moreover, substantial evidence from studies in patients with temporal lobe epilepsy (TLE) demonstrated that frontal lobe dysfunction has been implicated in altered states of awareness and inattention.^37^, ^38^

## 5 LIMITATIONS

There are a few limitations that should be noted. First, this is a retrospective study in a tertiary medical center, including all patients with AE who fulfilled the criteria and underwent ^18^F‐FDG PET/CT at the acute stage. The single‐center and retrospective properties of the study may have potential selection bias. Moreover, due to the retrospective nature of this study, no sample size calculation was performed. Second, our study regarded AE as a whole, regardless of their antibody status. Further comparisons between AE with different antibodies could not be made due to the relatively small subgroup of AE with specific antibody status. Third, it is a pity that those patients did not undergo re‐examination of brain PET after the acute stage, leading to the inability to monitor brain metabolic alterations dynamically. Last, the findings from our multivariate Cox regression model need to be further validated in future research with a larger sample size. Additionally, the usage of sedatives and corticosteroids should be noted. Although our enrolled patients were required to discontinue medicine 24 h before scanning, some researchers still mentioned the effects of corticosteroids and sedatives on cerebral metabolism.^39^ Therefore, further prospective, multicentric studies involving larger sample sizes should pay more attention to this issue.

## 6 CONCLUSION

In conclusion, this study revealed brain metabolic patterns in a cohort of patients with AE. ^18^F‐FDG PET/CT was more sensitive than brain MRI in the early stage of AE. A mixed metabolic pattern, characterized by large areas of cortical hypometabolism with focal hypermetabolism was a general metabolic pattern. Reduced level of consciousness was independently associated with poor outcome of AE. Decreased metabolism of the right superior frontal gyrus along with increased metabolism of the middle and upper brainstem may predict a poor long‐term prognosis. Further prospective, multicentric studies involving larger sample sizes will be warranted to confirm the role of ^18^F‐FDG PET/CT in the diagnostic performance and predictive value in patients with AE.

## CONFLICT OF INTEREST STATEMENT

The authors declare that they have no conflicts of interest.

## CONSENT FOR PUBLICATION

Written informed consent for participation and publication was obtained from involved patients or their legal guardians.

## Supporting information


Data S1:

## Data Availability

The datasets generated during and/or analyzed during the current study are available from the corresponding author upon reasonable request.
