# Supplementary material for: The clinical and predictive value of 18F‐FDG PET/CT metabolic patterns in a clinical Chinese cohort with autoimmune encephalitis
Source: CNS Neurosci Ther. 2024 Jul 1;30(7):e14821. doi: 10.1111/cns.14821 (PMC11215490; doi:10.1111/cns.14821)

Supplemental Table1: The clinical, laboratory and radiological features of patients with possible autoimmune encephalitis (AE) and probable autoimmune encephalitis (AE)

| No./ classification | Main clinical presentations | CSF profiles | Brain MRI | Immunotherapy protocols | Tumor | Response to treatment | Long-term outcome | CASE at the last  follow-up |
| --- | --- | --- | --- | --- | --- | --- | --- | --- |
| No.2/ possible- AE | Lethargy; psychiatric symptoms;  refractory seizures | 7 cells/mm^3^  OCBs | Normal | IVMP, PLEX, RTX | No | Yes | Good | 2 |
| No.3/ possible- AE | Fever;  status epilepticus;  psychiatric symptoms;  autonomic dysfunction | 3 cells/ mm^3^  OCBs | Normal | IVMP, PLEX, IVIG | No | Yes | Good | 2 |
| No.4/ probable-AE | Behavioral abnormalities;  ataxia | 8 cells/ mm^3^ | Multifocal white matter  lesions involving the brainstem, cerebellum, frontal lobes | IVIG | No | Yes | Good | 0 |
| No.7/ probable-AE | Decreased level of consciousness;  behavioral changes;  central hypoventilation | 8 cells/ mm^3^ | Bilateral cerebral  hemispheres,  brainstem, basal  ganglia and right  thalamus signal  abnormality | IVIG | Colon cancer | No | Died | NA |
| No.10/possible-AE | Refractory seizure;  memory  deficit;  speech dysfunction | 4 cells/ mm^3^  OCBs | Normal | IVIG | No | No | Bad | 5 |
| NO.13/possible-AE | Behavioral changes;  memory impairment | 3 cells/ mm^3^  OCBs | Normal | IVIG | Lung cancer | No | Died | NA |
| No.14/possible-AE | Behavioral changes;  memory impairment;  speech dysfunction | 6 cells / mm^3^ | Normal | IVIG | No | Yes | Bad | 8 |
| No.15/probable-AE | Status epilepticus;  behavioral changes | OCBSs | Right lateral  ventricle and right  frontoparietal  signal abnormality  with focal cerebral  atrophy | IVMP, IVIG | No | Yes | Good | 1 |
| No.25/probable-AE | Confusion;  behavioral changes | 8 cells / mm^3^ | Bilateral basal  ganglia, cerebral  peduncle and  thalamus signal  abnormality | IVMP | No | No | Bad | 4 |
| No.29/probable-AE | Refractory seizure;  decreased level of consciousness | 26 cells / mm^3^  OCBs | Increased T2/FLAIR signal in bilateral MTL, frontal lobes | IVIG | No | Yes | Good | 2 |
| No.30/ probable-AE | Decreased level of consciousness;  central hypoventilation;  ataxia | OCBs | Medulla oblongata  T2/FLAIR signal  abnormality | IVIG | No | No | Died | NA |

***Abbreviations:*** *MRI, magnetic resonance imaging; CSF, cerebrospinal fluid; F, female; M, male;T2/FLAIR, T2 weighted/fluid-attenuated inversion recovery; MTL, medial temporal lobe; BG, basal ganglia; CASE,* *Clinical Assessment Scale in Autoimmune Encephalitis; OCBs, Oligoclonal bands;* *IVMP, intravenous methylprednisolone pulse; IVIG, intravenous immunoglobulin; PLEX, plasma exchange; RTX, rituximab.*

Supplemental Table 2. The details of cerebral MRI imaging results and brain ^18^F-FDG PET/CT metabolic patterns based on SPM for individual patients with autoimmune encephalitis

| Patient/ classification | Main clinical symptoms | Autoantibody | Time from disease onset to MRI (days) | Cerebral MRI imaging result | Time from disease onset to ^18^F-FDG PET/CT (days) | Interval form MRI and ^18^F-FDG PET/CT (days) | PET results based on SPM  Hypermetabolism Hypometabolism | |
| --- | --- | --- | --- | --- | --- | --- | --- | --- |
| No.1/definite-AE | Dystonia;  seizures | D2R | 80 | Increased T2/FLAIR signal in the right hippocampal region | 93 | 13 | Parietal lobe  Cerebellar vermis  Midbrain  BG | Parietal lobe  Frontal lobe |
| No.2/ possible- AE | Lethargy; psychiatric symptoms;  refractory seizures | Negative | 6 | Normal | 10 | 4 | BG  Temporal lobe  Frontal lobe | Frontal lobe  Limbic lobe  Cingulate gyrus |
| No.3/ possible- AE | Fever;  status epilepticus;  psychiatric symptoms;  autonomic dysfunction | Negative | 28 | Normal | 31 | 3 | Parietal lobe  Brainstem  BG | Frontal lobe  Limbic lobe  Cingulate gyrus  Insular lobe  Parietal lobe |
| No.4/ probable-AE | Behavioral abnormalities;  ataxia | Negative | 20 | Multifocal white matter  lesions involving the brainstem, cerebellum, frontal lobes | 31 | 9 | Limbic lobe  Parietal lobe | Frontal lobe  Parietal lobe |
| No.5/anti-NMDAR encephalitis | Memory deficit;  ataxia | NMDAR | 33 | Normal | 35 | 2 | Temporal lobe  Frontal lobe | Caudate  Limbic lobe  MTL |
| No.6/ definite-AE | Seizures;  psychiatric symptoms | GABA_B_R | 14 | Normal | 20 | 6 | Midbrain  MTL  BG  Cerebellum | Frontal lobe  Parietal lobe  Limbic lobe |
| No.7/ probable-AE | Decreased level of consciousness;  behavioral changes;  central hypoventilation | Negative | 8 | Bilateral cerebral hemispheres, brainstem, basal ganglia and right thalamus signal abnormality | 10 | 2 | Cerebellum  Brainstem  MTL  BG  Parietal lobe | Frontal lobe  Parietal lobe  Limbic lobe  Insular lobe |
| No.8/ definite-AE | Seizures;  psychiatric symptoms | GABA_B_R | 83 | Normal | 88 | 5 | BG  MTL  Brainstem  Insular lobe | Frontal lobe  Temporal lobe  Parietal lobe  Occipital lobe |
| No.9/ definite-AE | Refractory seizures;  psychiatric symptoms | SOX1 | 22 | Normal | 24 | 2 | Brainstem  Temporal lobe  Limbic lobe  BG | Frontal lobe  Parietal lobe  Occipital lobe  Limbic lobe |
| No.10/ possible-AE | Refractory seizure;  memory  deficit;  speech dysfunction | Negative | 10 | Normal | 14 | 4 | Temporal lobe  Parietal lobe | Frontal lobe  Parietal lobe |
| No.11/ definite-AE | Memory impairment;  fever;  lethargy | CASPR2 | 12 | Normal | 17 | 5 | Cerebellum  Temporal lobe  Parietal lobe | Frontal lobe  Parietal lobe  Insular lobe |
| No.12/ anti-NMDAR encephalitis | Seizures;  central hypoventilation | NMDAR | 13 | Increased T2/FLAIR signal abnormalities in the regions of right midbrain and  right hippocampus | 25 | 12 | MTL  BG  Cerebellum | Frontal lobe  Parietal lobe  Occipital lobe |
| No.13/possible-AE | Behavioral changes;  memory impairment | Negative | 21 | Normal | 23 | 2 | Cerebellum  Brainstem  Limbic lobe  Parietal lobe | Frontal lobe  Parietal lobe  Limbic lobe |
| No.14/ possible-AE | Behavioral changes;  memory impairment;  speech dysfunction | Negative | 29 | Normal | 33 | 4 | Occipital lobe  Brainstem  Cerebellum  BG | Frontal lobe  Temporal lobe  Parietal lobe  Limbic lobe |
| No.15/ probable-AE | Status epilepticus;  behavioral changes | Negative | 13 | Right lateral ventricle and right frontoparietal signal abnormality with focal cerebral atrophy | 15 | 2 | Frontal lobe  Parietal lobe  Limbic lobe  BG  Temporal lobe  Insular lobe | Frontal lobe  Thalamus  Insular lobe |
| No.16/Ab-positive-LE | Seizure;  behavioral changes | GABA_B_R | 47 | Increased T2/FLAIR signal in bilateral MTL | 50 | 3 | Limbic lobe  MTL  BG  Brainstem  Cerebellum | Frontal lobe  Parietal lobe  Occipital lobe |
| No.17/definite-AE | Behavioral changes;  dystonia | CASPR2 | 12 | Normal | 15 | 3 | Temporal lobe  Limbic lobe  Parietal lobe  Insular lobe | Frontal lobe  Parietal lobe |
| No.18/definite-AE | Seizure;  behavioral changes | IgLON5 | 14 | Normal | 17 | 3 | Cerebellum  Parietal lobe  Frontal lobe | Frontal lobe  Parietal lobe  Temporal lobe  Limbic lobe  Occipital lobe |
| No.19/ definite-AE | Seizure;  behavioral changes | SOX1 | 15 | Normal | 18 | 3 | -- | -- |
| No.20/ Ab-positive-LE | Seizure;  behavioral changes | GABA_B_R | 17 | Increased T2/FLAIR signal in bilateral MTL | 20 | 3 | MTL  Limbic lobe  Cerebellum  Brainstem | Frontal lobe |
| No.21/ definite-AE | Status epilepticus;  decreased levels of consciousness | LGI-1 | 38 | Normal | 43 | 5 | Cerebellum  Brainstem  MTL  Limbic lobe  BG | Frontal lobe  Parietal lobe  Occipital lobe |
| No.22/anti-NMDAR encephalitis | Seizures | NMDAR | 14 | T2/FLAIR hyperintense lesions in bilateral frontal lobes | 17 | 3 | Limbic lobe  Frontal lobe  Temporal lobe  Occipital lobe  Cerebellum  Brainstem | Occipital lobe  Parietal lobe |
| No.23/ anti-NMDAR encephalitis | Seizure;  behavioral changes | NMDAR | 22 | Normal | 25 | 3 | Parietal lobe  Cerebellum | Temporal lobe  Frontal lobe |
| No.24/ anti-NMDAR encephalitis | Seizure;  behavioral changes | NMDAR | 10 | Normal | 11 | 1 | Cerebellum  Brainstem  Temporal lobe  Insular lobe | Occipital lobe  Limbic lobe  Parietal lobe |
| No.25/probable-AE | Confusion;  behavioral changes | Negative | 25 | Bilateral basal ganglia, cerebral peduncle and thalamus signal abnormality | 27 |  | Brainstem  Cerebellum  Temporal lobe | Frontal lobe  Parietal lobe  Limbic lobe |
| No.26/ anti-NMDAR encephalitis | Psychiatric symptoms;  Decreased levels of consciousness | NMDAR | 7 | Normal | 10 |  | -- | -- |
| No.27/definite-AE | Decreased level of consciousness;  central hypoventilation | GAD65 | 12 | Normal | 14 |  | BG  Cerebellum  Brainstem  Occipital lobe | Temporal lobe |
| No.28/ anti-NMDAR encephalitis | Fever;  seizure;  decreased level of consciousness | NMDAR | 51 | Normal | 55 |  | Parietal lobe  Temporal lobe  Cerebellum  Brainstem  Limbic lobe | Frontal lobe |
| No.29/probable-AE | Refractory seizure;  decreased level of consciousness | Negative | 7 | Increased T2/FLAIR signal in bilateral MTL, frontal lobes | 13 |  | BG | Frontal lobe  Parietal lobe  Limbic lobe |
| No.30/ probable-AE | Decreased level of consciousness;  central hypoventilation;  ataxia | Negative | 33 | Medulla oblongata T2/FLAIR signal abnormality | 36 |  | Cerebellum  Frontal lobe  BG  Brainstem  Limbic lobe | Limbic lobe  Frontal lobe  Temporal lobe  Parietal lobe  Occipital lobe |
| No.31/ anti-NMDAR encephalitis | Central hypoventilation;  decreased level of consciousness | NMDAR | 49 | Normal | 55 |  | Cerebellum  Parietal lobe  BG | Parietal lobe  Limbic lobe |
| No.32/ anti-NMDAR encephalitis | Behavioral changes;  autonomic dysfunction | NMDAR | 80 | Normal | 83 |  | Brainstem  Cerebellum  Limbic lobe  Temporal lobe | Frontal lobe  Parietal lobe  Occipital lobe |
| No.33/definite-AE | Seizure | GABA_B_R | 7 | Normal | 10 |  | Temporal lobe  BG  Limbic lobe  Cerebellum  Brainstem | Frontal lobe  Parietal lobe |
| No.34/ Ab-positive-LE | Memory deficit;  seizures | LGI-1 | 23 | Increased T2/FLAIR signal in bilateral MTL | 26 |  | MTL  Temporal lobe  Limbic lobe  Cerebellum  Frontal lobe  Parietal lobe  Brainstem | Frontal lobe  Occipital lobe |
| No.35/ anti-NMDAR encephalitis | Dystonia;  Decreased levels of consciousness | NMDAR | 14 | Right temporal lobe and bilateral cerebellar hemispheres signal abnormality | 18 |  | BG  Limbic lobe  Brainstem  Temporal lobe  Cerebellum  Insular lobe | Frontal lobe  Occipital lobe  Parietal lobe |

***Abbreviations:*** *AE, autoimmune encephalitis;* *Ab, antibody; LE, limbic encephalitis;* *MRI, magnetic resonance imaging; ^18^F-FDG PET/CT, 2-deoxy-2-18Ffluoro-D-glucose-(^18^F-FDG) positron emission tomography/computed tomography; SPM, Statistical Parametric Mapping; F, female; M, male; NMDAR, N-methyl-D-aspartate receptor; GABA_B_R, Gamma-aminobutyric acid type B receptor; CASPR2, contactin-associated protein-2; LGI-1, leucine-rich glioma inactivated 1; D2R, dopamine D2 receptor; IgLON5, IgLON family member 5; GAD65, glutamic acid decarboxylase 65; T2/FLAIR, T2 weighted/fluid-attenuated inversion recovery; MTL, medial temporal lobe; BG, basal ganglia.*

Supplemental figure 1. The brain metabolism patterns of 10 patients with anti-NMDAR encephalitis based on SPM, in comparison to healthy controls (n=43) (*p*<0.05, FWE corrected). Slice view (a) and render view (b) were created by xjView toolbox (https://www.alivelearn.net/xjview/). The hypermetabolism regions were shown in orange-red color, and hypometabolism regions were shown in blue-green color.

Annotation: ^18^F-FDG PET/CT, 2-deoxy-2-^18^Ffluoro-D-glucose-(^18^F-FDG) positron emission tomography/computed tomography; SPM, Statistical Parametric Mapping; AE, autoimmune encephalitis; FWE, family-wise error.


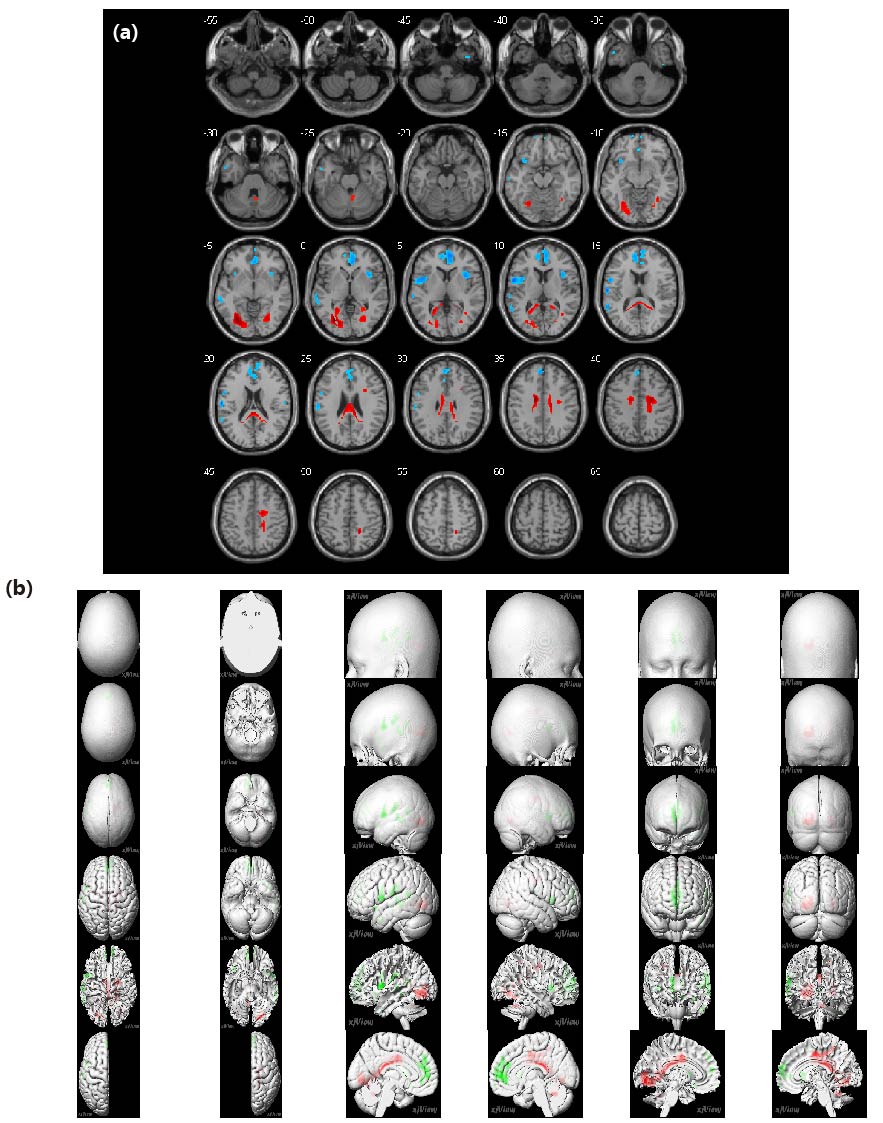

Supplement: Supplementary file 1 — Data S1: [file CNS-30-e14821-s001.docx]
